# Multi‐Omics Analysis Reveals *TO* Gene's Association With Food Selection and Lifespan in Minor‐Worker Ants Post‐Queen Loss

**DOI:** 10.1002/ece3.71508

**Published:** 2025-06-07

**Authors:** Jun Huang, Shizhen Wang, Chendi Yu, Hongli Su, Zhitao Jiang, Xiaowei Li, Yaobin Lu, Juan Zhang

**Affiliations:** ^1^ State Key Laboratory for Quality and Safety of Agro‐Products Institute of Plant Protection and Microbiology, Zhejiang Academy of Agricultural Sciences Hangzhou China; ^2^ Institute of Garden Plants and Flowers Zhejiang Academy of Agricultural Sciences Hangzhou China; ^3^ School of Chemistry and Molecular Biosciences, Australian Infectious Diseases Research Centre The University of Queensland Brisbane Queensland Australia; ^4^ College of Forestry and Biotechnology Zhejiang A&F University Hangzhou China; ^5^ College of Biological Sciences University of California Oakland California USA; ^6^ Institute of Bio‐Interaction Xianghu Laboratory Xiaoshan China

**Keywords:** food selection, longevity, queen loss, *Solenopsis invicta*, takeout gene, work ant

## Abstract

The queen ant is central to colony reproduction and chemical communication. Removing her causes significant phenotypic changes, such as wing loss and oviposition in virgins, and the emergence of reproductive pseudoqueens. However, the effects of queen loss in the workers of invasive *Solenopsis invicta* have not been thoroughly documented. Our study compares worker behavior and gene expression post‐queen removal, revealing altered dietary preferences, life expectancy, and no change in necrophoresis behavior. The queen's absence mildly affects gene expression in major workers, but TO and MRJP1 expression rise in minors. Metabolite changes relate to unsaturated fatty acid biosynthesis and autophagy, with increased lysosome production in queenless minors, likely due to the phosphatidylinositol signaling pathway in response to changes in nutritional status. This upregulation may clear cellular debris, ensuring nutrient stability and cellular integrity, and activate the PI3K/Akt pathway to regulate FoxO, enhancing resilience to sugar intake reduction and promoting longevity. We also suggest that the metabolite 12(Z), 15(Z)‐heneicosadienoic acid is key in MRJP synthesis, linked to worker ant longevity.

## Graphical abstract

Queenless minor workers diminish sugar intake and extend their lifespan. The expression of TO and MRJP1 notably escalated in minor workers with queen loss. Gene TO may trigger pathways in the biosynthesis of unsaturated fatty acids and autophagy. TO and MRJP1 may jointly participate in the synthesis of MRJPs and contribute to the life extension of minor workers.

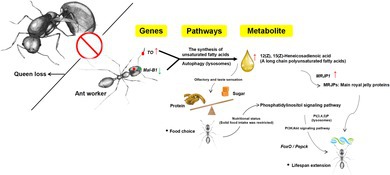

## 1 Introduction

Social insects, including ants and bees, exhibit remarkable characteristics of communal living and a well‐defined division of labor (Wilson 1971; Robinson et al. 2005). Within these societies, thousands of individuals operate with high organization and coordination, facilitated by pheromones secreted by specialized glands. These pheromones are categorized into releaser and primer types (Karlson and Burtenandt 1959; Alaux et al. 2010). Releaser pheromones prompt immediate behavioral responses, such as the alarm pheromone emitted during an attack (Roberts et al. 2011) or the trail pheromone during foraging (Suckling et al. 2012). In contrast, primer pheromones exert long‐term effects, subtly altering the recipient's behavior through physiological and developmental changes, exemplified by the queen pheromone (Vargo 1992; Klobuchar and Deslippe 2002). In fire ants, the queen pheromone not only inhibits wing shedding and ovary development in female alates of the same species (Fletcher and Blum 1981; Burns et al. 2005) but also suppresses oviposition by rival queens (Vargo 1992). The absence of the queen leads to ovarian degeneration or loss of egg‐laying function in workers, a phenomenon confirmed in honey bees (Hoover et al. 2003; Cardoso‐Júnior et al. 2021). However, the impact on worker ants remains largely unknown.

Employing the ant *Harpegnathos saltator* as a model for studying behavioral plasticity, Peeters et al. (2000) and Bonasio et al. (2010) revealed that the absence of a queen could induce worker ants to become gamergates, accompanied by an increase in lifespan, alterations in gene expression, and changes in brain cellular composition and neurohormonal profiles (Penick et al. 2014; Gospocic et al. 2017; Sheng et al. 2020; Yan et al. 2022). Utilizing CRISPR/Cas9 technology for ant gene knockout, Gospocic et al. (2021) further elucidated the molecular mechanisms underlying the workers' transition to queens, identifying the transcription factor *Kr‐h1* as a key regulator of social hierarchy boundaries. Workers of two independently derived queenless *Temnothorax* ant species have been observed to develop their ovaries, commence egg‐laying, extend their lifespan, and exhibit distinct gene expression in the fat body (Kohlmeier et al. 2017; Negroni et al. 2020, 2021). Histone acetylation may also play a significant role in the molecular mechanisms underlying worker reproduction (Choppin et al. 2021). This epigenetic modification can change with variations in nutritional conditions or levels, thereby further influencing the expression of genes related to lifespan or behavior (Weaver et al. 2023). While the transcriptomic factors for reproduction have been described, how to distinguish them from other factors, such as queen pheromone absence, extended lifespan, and nutritional changes, has not yet been detailed in the literature.

Global gene expression changes have been identified in virgin queens of fire ants that detect the loss of the mother queen and initiate competition for reproductive dominance (Wurm et al. 2010). Additionally, Manfredini et al. (2014) discovered that the colony's condition (queenless vs. queenright) might influence molecular pathways related to worker task performance, including lipid, carbohydrate, protein, and energy metabolism levels. However, the subtle or potential trends in gene expression related to reproduction and longevity in fire ant workers post‐queen loss are not apparent, given numerous reports of the absence of spermatheca or ovarioles in the abdomen of *Solenopsis invicta* workers (Bourke 1988; Aron et al. 1995; Manfredini et al. 2014; Villalta et al. 2018; Hoffmann et al. 2024). Here, we focus on the invasive fire ant *S. invicta* , emerging as a novel model system in sociogenomics, with a sequenced genome and a comprehensive understanding of biological ecology, organizational behavior, and population regulation mechanisms (Wurm et al. 2010; Wang et al. 2013; Manfredini et al. 2014). We selected two worker groups of different sizes and ages: minor workers, typically older individuals engaged in foraging, and major workers, generally younger individuals within the nest (Cassill and Tschinkel 1999). Employing behavioral and survival data alongside molecular biotechnology, we address the following questions: (i) Queen ants and worker ants exhibit brain specialization in different directions and to varying degrees. The former indirectly regulates the social behaviors of worker ants, including corpse removal and cleaning, potentially through pheromones (Li et al. 2022). Therefore, do worker ant‐containing colony fragments still maintain normal efficiency in necrophoresis and cleaning behaviors after a prolonged queenless period (25 days)? (ii) Changes in queen pheromones may lead to alterations in worker ant behaviors, including food selection behavior. This is because pheromones coordinate social cooperation and behavioral responses by inducing a wide range of physiological and behavioral reactions (Yan and Liebig 2021). Does the removal of the queen alter the food selection and carrying habits of these ants, and in what direction and to what extent? (iii) What is the mortality rate of worker ants after 45 days, and is there a change in lifespan? (iv) Do gene expression levels related to reproduction and metabolism change in workers following queen loss? We hypothesize that necrophoresis and cleaning behaviors, longevity, and gene expression levels will differ between the two treatment groups (queenright vs. queenless). Even without the development of ovaries and the transformation of reproductive individuals, *S. invicta* workers may exhibit changes at the transcriptome level of reproductive genes under prolonged environmental selection pressure. Understanding the reproductive remodeling capacity in worker ants post‐queen loss is crucial for guiding the monitoring of their colonization risks.

## 2 Materials and Methods

### 2.1 Ant Collection, Rearing and Sampling

Twelve different colonies of polygyne *S. invicta* were collected from Jinhua, Zhejiang Province, China (29.08°N, 119.69°E, altitude 52.92 m; habitat: park green belt) in late November 2022. The ants were transported to the laboratory in plastic containers with Fluon‐coated internal walls to prevent escape (see Figure S2 in the Supporting Information for a detailed description). Upon arrival, the colonies were acclimated and reared under standard laboratory conditions for 1 month prior to sampling, following the artificial diet protocols reported by Banks et al. (1981). The polygyne social structure of *S. invicta* was verified by the presence of multiple queens per colony and the proximity (< 5 m) of nests within a 100 m^2^ area, as per Porter (1992).

**FIGURE 1:**
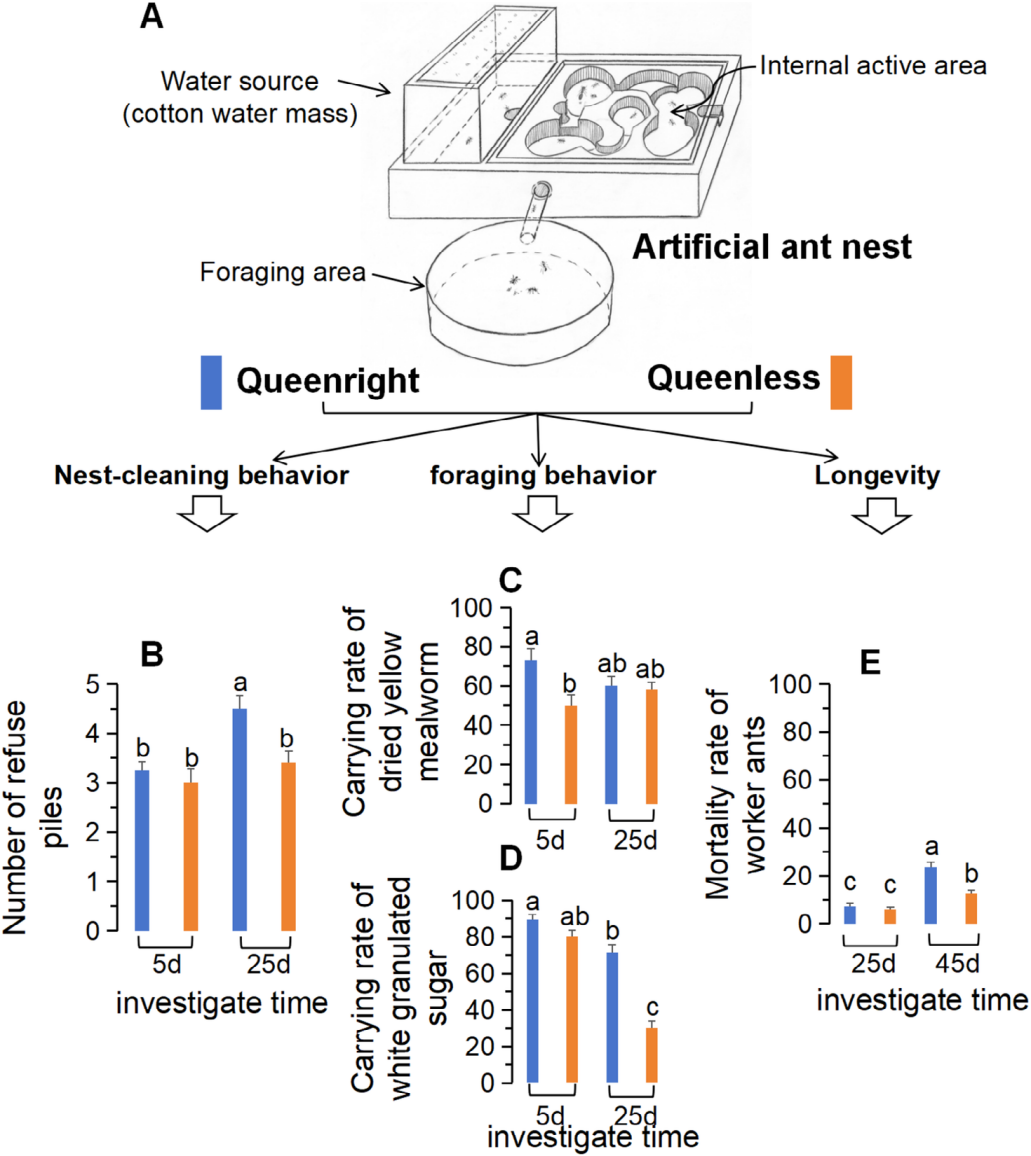
Behavioral and longevity observations of *Solenopsis invicta* workers in queenright and queenless colonies. A diagram of an artificial ant nest is depicted, highlighting the internal activity area, water source area, and foraging area (A). Behavioral observations, including refuse pile count (B), carrying rates of dried yellow mealworms (C), and white granulated sugar (D), were recorded on days 5 and 25, and 12 independent biological replicates; the carrying rate was measured in a time unit of 5 days. Longevity was assessed by counting deceased workers on days 25 and 45 (E), 5 independent biological replicates. Statistical significance between groups is denoted by different lowercase letters (*p* < 0.05) using Tukey's Honest Significant Difference (HSD) test.

For the behavioral observation experiment, two colony fragments were sampled from each of the 12 colonies: one queenright (QR) fragment containing a queen, two winged virgin queens, and at least 300 workers (with an equal division of minor and major workers); and one queenless (QL) fragment comprising approximately 300 workers (also equally divided). The ants were placed in artificial nests constructed from plaster and topped with a transparent glass cover (see Figure 1A and Figure S1 in the Supporting Information for a detailed description). Behavioral activities were monitored continuously for 25 days, while mortality was recorded over a 45‐day period.

Sampling for molecular biology analysis occurred in two phases. In the initial phase (In), prior to the ants' transfer to the artificial nest, samples were taken from two QR groups: one of minor workers (QRMi‐In) and one of major workers (QRMa‐In). Minor workers, typically older and engaged in foraging, were collected using ham sausage slices as bait. Major workers, generally younger and from within the mound, were collected using a barbecue skewer to encourage them to climb up, as described by Cassill and Tschinkel (1999). In the after phase (Af), 25 days post‐transfer to the artificial nest, samples were taken from four groups: QR minor and major workers (QRMi‐Af and QRMa‐Af, respectively) and QL minor and major workers (QLMi‐Af and QLMa‐Af, respectively). Three samples randomly from different fragments in the minor or major worker groups were used for transcriptome sequencing and metabolomics analysis, respectively. Each sample or group consisted of 30 worker ants from the same original colony. Minor workers were collected from foraging and water source areas, while major workers were gathered from the internal active area by opening the top glass lid and using a brush. Colony fragments were maintained under the same conditions as previously described.

### 2.2 Ant Behavior and Longevity Observations

Behavioral observations of workers in both queenright and queenless groups were conducted on the 5th and 25th days post‐setup. The behaviors recorded included:

1. Refuse pile formation: The number of refuse piles was recorded, defined as any food fragments, debris from feeding, or deceased conspecifics neatly stacked in the nest's corner (see Figure S1 in the Supporting Information for a detailed description).
2. Food transportation: The movement of food items (dried yellow mealworms and white granulated sugar) to the internal activity area was monitored. On days 1, 10, and 20, five dried yellow mealworms and 20 sugar cubes were placed in the foraging area. The cotton water mass was refreshed every 2 days. Successful food transportation was scored when the mealworms were dismembered and partially consumed, with the remaining parts deposited in the refuse pile (see Figure S1 in the Supporting Information for a detailed description).
3. Mortality count: The number of deceased workers (corpses) was recorded on days 25 and 45, both within refuse piles and other areas of the nest. If the corpses have already been dismembered, the number of corpses should be counted by their heads or abdomens. Since it was difficult to distinguish between the corpses of minor and major workers, there was no further classification for statistical purposes here. Dead individuals on the first day were excluded from the statistics to avoid the influence of ant deaths caused by human operations during the initial stage.

The first two behavioral observations were replicated 12 times to ensure data reliability, while the mortality count was replicated 5 times to assess longevity trends, using an ant colony as a bioassay.

### 2.3 Transcriptome Analysis

#### 2.3.1 RNA Isolation and Library Preparation

Total RNA was extracted from *S. invicta* using the mirVana miRNA Isolation Kit (QIAGEN) following the manufacturer's protocols. RNA yield was quantified using a NanoDrop 2000 spectrophotometer (Thermo Scientific, USA), and integrity was assessed via agarose gel electrophoresis with ethidium bromide staining. Libraries were prepared using the VAHTS Universal V6 RNA‐seq Library Prep Kit and sequenced on an Illumina NovaSeq 6000 platform, generating 150 bp paired‐end reads. Transcriptome sequencing and analysis services were provided by OE Biotech Co. Ltd. (Shanghai, China).

#### 2.3.2 RNA Sequencing and Differentially Expressed Genes Analysis

Approximately 49.29 million clean reads per sample were retained for further analysis. These reads were aligned to the reference genome using HISAT2 (Kim et al. 2015). The reference genome version is Sinv2.0, which is available in the GenBank database of NCBI (National Center for Biotechnology Information, USA). Gene expression levels were estimated by calculating the FPKM (Fragments Per Kilobase of transcript per Million mapped reads) (Roberts et al. 2011), and read counts were obtained using HTSeq‐count (Anders et al. 2015). PCA (Principal Component Analysis) was conducted using R (version 3.2.0) to assess sample reproducibility.

Differential gene expression analysis was performed using DESeq2. A *Q*‐value < 0.05 and a fold‐change threshold of either > 2 or < 0.5 were set to identify significantly differentially expressed genes (DEGs). Hierarchical cluster analysis of DEGs was conducted using R (version 3.2.0) to illustrate gene expression patterns across different groups and samples. A radar chart of the top 30 DEGs was created using the R package ‘ggradar’ to visualize the expression profiles of up‐ and down‐regulated genes.

Furthermore, functional enrichment analysis of DEGs was performed using hypergeometric distribution‐based methods to identify significantly enriched terms in GO (Gene Ontology), KEGG (Kyoto Encyclopedia of Genes and Genomes) pathways, Reactome, and WikiPathways, all conducted using R (version 3.2.0).

### 2.4 Fire Ant Tissues Metabolomics Analysis

#### 2.4.1 Sample Pretreatment

A combined approach of Gas Chromatography–Mass Spectrometry (GC–MS) and Liquid Chromatography‐Mass Spectrometry (LC–MS) was utilized to investigate differential metabolites in fire ant tissues. Thirty milligrams of each sample were weighed and transferred into a 1.5 mL Eppendorf tube, to which two small stainless steel balls were added. A 600 μL volume of extraction solvent (methanol/water, 4:1, v/v, containing L‐2‐chlorophenylalanine at 4 μg/mL) was added to each sample. Samples were then stored at −20°C for 2 min prior to grinding at 60 Hz for 2 min. Following grinding, samples were sonicated for 30 min in an ice‐water bath and subsequently cooled at −40°C for 30 min. After cooling, samples were centrifuged at 4°C at 12,000 rpm for 10 min. Then, 150 μL of the supernatant was transferred to a glass vial and dried using a freeze concentration centrifugal dryer, then stored at −80°C until analysis for LC–MS analysis. For GC–MS analysis, 80 μL of 15 mg/mL methoxylamine hydrochloride in pyridine was added to the dried samples, vortexed for 2 min, and incubated at 37°C for 90 min. Subsequently, 50 μL of BSTFA (containing 1% TMCS) and 20 μL of n‐hexane were added to the mixture, vortexed for 2 min, and then derivatized at 70°C for 60 min. After derivatization, samples were allowed to equilibrate at room temperature for 30 min before proceeding to GC–MS analysis.

#### 2.4.2 Sample Treatment and Data Analysis

LC–MS Analysis: The metabolic profiling was performed using an ACQUITY UPLC I‐Class system (Waters Corporation, Milford, USA) interfaced with a VION IMS QTOF Mass Spectrometer (Waters Corporation, Milford, USA). The system operated in both ESI positive and negative ion modes. Raw LC–MS data were processed utilizing Progenesis QI V2.3 software (Nonlinear, Dynamics, Newcastle, UK). Differential metabolites were identified based on VIP (Variable Importance in Projection) values greater than 1.0 and *p*‐values less than 0.05.

GC–MS Analysis: Derivatized samples were analyzed using an Agilent 7890B gas chromatography system coupled to an Agilent 5977A MSD system (Agilent Technologies Inc., CA, USA). Separation of the derivatives was achieved with a DB‐5MS fused‐silica capillary column (30 m ×0.25 mm × 0.25 μm, Agilent J & W Scientific, Folsom, CA, USA). Raw GC/MS data (.D format) were converted into analysis basic files. Differential metabolites were selected applying the same criteria as for LC–MS, with VIP values greater than 1.0 and *p*‐values less than 0.05.

### 2.5 Validation of Differential Expression of Candidate Genes Using Quantitative Real‐Time PCR

The expression levels of 11 candidate genes involved in biological processes were validated using quantitative real‐time PCR (RT‐PCR) on a LightCycler 480 II Real‐time PCR Instrument (Roche, Swiss). EF1‐beta was chosen as the internal reference gene, with primer sequences detailed in Table S1. Expression levels were measured in queen‐right and queenless groups of *S. invicta* minor workers, with three replicates per group.

### 2.6 Statistical Analysis

Data normality and homogeneity of variance were assessed using Shapiro–Wilk tests for ant behavioral and physiological tests. Data meeting the criteria for normal distribution and homogeneity were analyzed further. Where necessary, data were normalized using square root or logarithmic transformations. Two‐way ANOVA was employed to compare the number of refuse piles, the carrying rate of dried yellow mealworm and white granulated sugar, and the mortality rate of dead workers between queenright and queenless groups, as well as across treatment times. The carrying rate referred to the proportion of the quantity carried on average within 5 days to the total quantity. Post hoc multiple comparisons were conducted using Tukey's HSD (Honestly Significant Difference) tests. mRNA expression levels of selected genes were normalized and calculated using the 2^−ΔΔCt^ method (Livak and Schmittgen 2001). Paired *t*‐tests were used to evaluate differences in gene expression between queenright and queenless groups. All statistical analyses were performed using SPSS version 14.0 (SPSS Inc., Chicago, IL, USA), with data presented as means ± standard errors.

## 3 Results

### 3.1 Ant Behavior and Longevity in Response to Queenright or Queenless

Necrophoresis behavior plays a vital role in the removal of conspecific corpses and the defense against harmful pathogens within the nest (Diez et al. 2013). In our study, refuse piles of *S. invicta* were observed to contain food remnants, dead bodies, and even fragments of cotton wool from the water source. Over time, there was a significant increase in the number of refuse piles in queenright colonies (Figure 1B, *F*_1,44_ = 7.78, *p* = 0.0078), with workers continuously establishing new piles within the confined space. In contrast, the number of refuse piles in queenless colonies did not significantly change, and scattered corpses were predominantly observed in the foraging area during the later stages of the investigation.

Regarding food transportation, a higher rate of dried yellow mealworms was noted in queenright colonies, but the carrying rate in queenless colonies also showed an increase in the later period (Figure 1C, *F*_1,44_ = 5.20, *p* = 0.028). Both time and treatment (the presence of the queen) significantly influenced the carrying rate of white granulated sugar (Figure 1D, *F*_1,44_ = 25.30, *p* < 0.001), with a notable decrease in the carrying rate in queenless colonies during the later stage. After a 45‐day observation period, the mortality rate of worker ants in queenright colonies significantly increased, nearly doubling compared to the queenless condition (Figure 1E, *F*_1,44_ = 10.74, *p* = 0.0047).

### 3.2 Differential Expression Genes in Response to Queenright or Queenless in Major and Minor Workers

**FIGURE 2:**
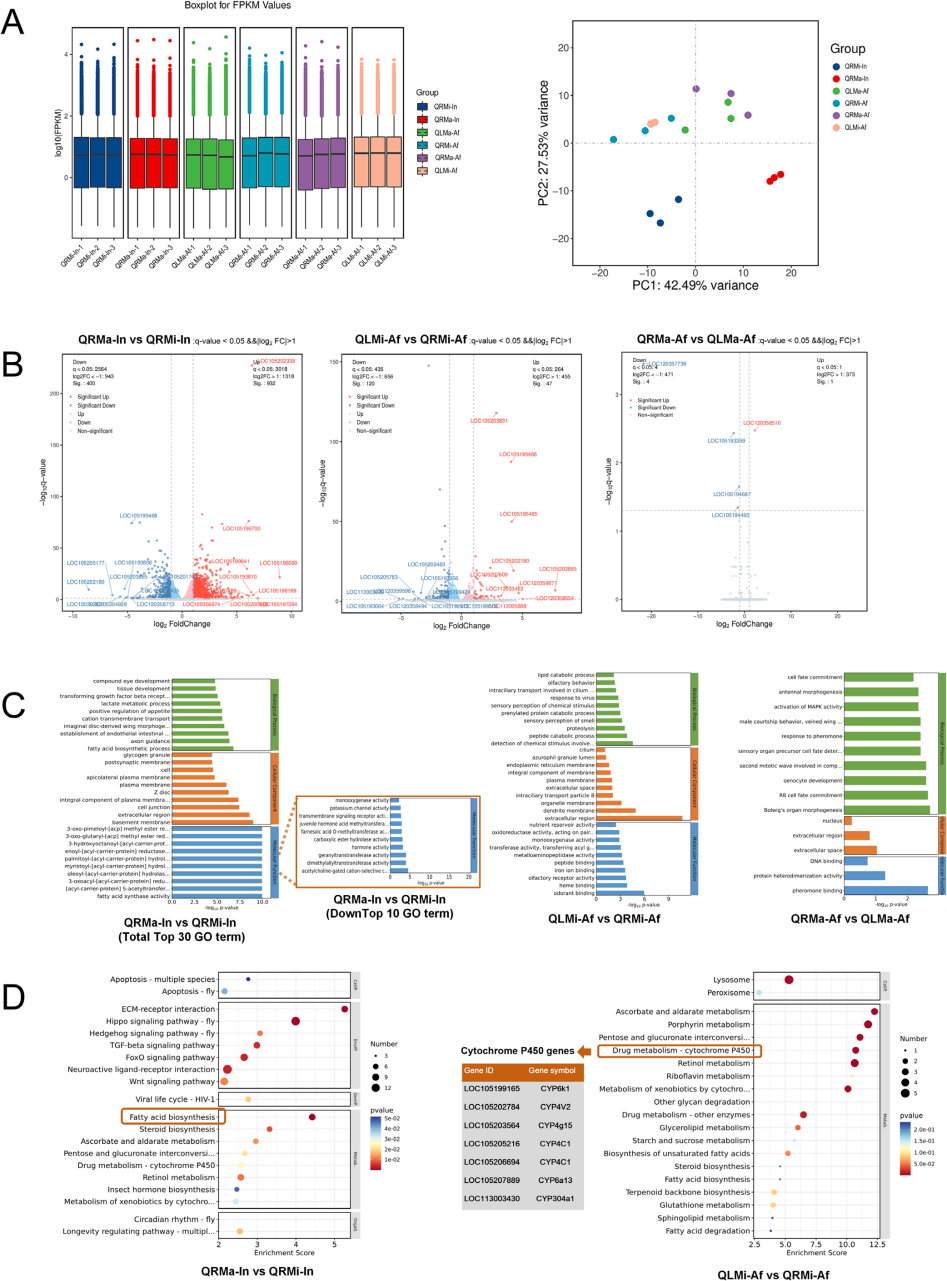
Transcriptome analysis of ant workers in queenless and queenright colonies. This figure presents a boxplot of FPKM values and a PCA scoring plot for each group (A). The differential expression of genes is illustrated in a volcano plot (B). The top 30 GO terms, including the top 10 downregulated terms, for QRMa‐In versus QRMi‐In, QLMi‐Af versus QRMi‐Af, and QRMa‐Af versus QLMa‐Af are shown (C). The top 20 KEGG pathway enrichments for the same comparisons are displayed (D).

To explore the molecular responses of fire ant workers to the presence or absence of the queen, we conducted a comprehensive transcriptome profiling analysis using tissue samples collected at the study's endpoint. The analysis generated a total of 130.16 Gb of CleanData, with each group yielding 6.9–7.6 Gb, and 89.9%–94.8% of clean reads were successfully aligned to the reference genome. The gene expression count per sample averaged approximately 520,000, as determined by FPKM (fragments per kilobase of exon model per million mapped reads) (Figure 2A). Genes with a *q*‐value < 0.05 and a fold‐change threshold of either > 2 or < 0.5 were selected for further analysis. The study identified a significant number of regulated genes across three distinct comparisons among the six groups: QRMa‐In versus QRMi‐In, QLMi‐Af versus QRMi‐Af, and QRMa‐Af versus QLMa‐Af. Comparative analysis revealed that 932 genes were up‐regulated and 400 genes were down‐regulated in major versus minor workers within queenright colonies (QRMa‐In vs. QRMi‐In). In minor workers of queenless colonies compared to queenright (QLMi‐Af vs. QRMi‐Af), 47 genes were up‐regulated and 120 genes were down‐regulated. In major workers, 1 gene was up‐regulated and 4 genes were down‐regulated between queenless and queenright colonies (QRMa‐Af vs. QLMa‐Af) (Figure 2B). To validate the transcriptome results, six genes were selected for confirmation by RT‐PCR.

Gene Ontology (GO) analysis was performed to elucidate the biological functions of the differentially expressed genes (DEGs). The DEGs were categorized into various GO terms, encompassing biological regulation, cellular processes, metabolic processes, multi‐organism processes, regulation of biological processes, response to stimulus signaling, extracellular binding, cellular components, membranes, organelles, and catalytic activities. The top 30 regulated GO terms in each group were enriched across three categories: biological process, molecular function, and cellular component. At the biological process level, fatty acid biosynthesis and tissue development were up‐regulated, while lipid metabolic processes were down‐regulated in the QRMa‐In versus QRMi‐In group. Lipid transport and compound biosynthesis were up‐regulated, while proteolysis and peptide metabolic processes were down‐regulated in the QLMi‐Af versus QRMi‐Af group. MAPK activity and pheromone‐related terms were down‐regulated, with no significant up‐regulated GO terms in the QRMa‐Af versus QLMa‐Af group (Figure 2C). Cellular component analysis revealed that up‐regulated GO terms were associated with the membrane, while down‐regulated terms were linked to the extracellular space in the QRMa‐In versus QRMi‐In group. Similar patterns of down‐regulation were observed in the other two groups. At the molecular function level, fatty acid synthesis was up‐regulated, while hormone activity was down‐regulated in the QRMa‐In versus QRMi‐In group. Acyl‐CoA dehydrogenase, transferase activity, and lipid binding were up‐regulated, whereas olfactory receptor activity and odorant binding were down‐regulated in the QLMi‐Af versus QRMi‐Af group, suggesting a connection to olfactory signals. Pheromone binding was specifically down‐regulated in the QRMa‐Af versus QLMa‐Af group (Figure 2C).

It is particularly noteworthy that genes related to olfactory receptor activity and odorant binding were down‐regulated in queenless workers, while genes involved in Acyl‐CoA dehydrogenase, transferase activity, and lipid binding were up‐regulated in minor workers. Hormone activity‐related genes were differentially expressed between major and minor workers in queenright colonies, indicating intrinsic differences. Furthermore, the down‐regulation of olfactory signal‐related genes in queenless conditions and the up‐regulation of lipid synthesis or transport genes in the presence of the queen suggest a complex regulatory network associated with queen presence and reproductive status (Wurm et al. 2010).

### 3.3 Different Expression Genes on KEGG Pathway Analysis

To elucidate the association between differentially expressed genes (DEGs) and cellular pathways, we performed KEGG pathway enrichment analysis based on the KEGG database. DEGs were significantly enriched in 56 and 28 predicted KEGG pathways for QRMa‐In versus QRMi‐In and QLMi‐Af versus QRMi‐Af comparisons, respectively. The scarcity of differential genes between QRMa‐Af and QLMa‐Af groups resulted in no enrichment in related pathways. The top 20 enriched pathways are illustrated in Figure 2D. Notably, up‐regulated genes were enriched in pathways such as ECM‐receptor interaction and Hippo signaling (categorized under environmental information processing), and fatty acid biosynthesis (metabolism). Conversely, down‐regulated genes were predominantly found in cytochromes P450 (metabolism), porphyrin metabolism, and retinol metabolism pathways. Cytochromes P450, a metabolic enzyme class present in all aerobic organisms, was significantly down‐regulated in queenless minor workers. Seven distinct cytochromes P450 genes were identified, including CYP6k1, CYP4V2, CYP4g15, two variants of CYP4C1, CYP6a13, and CYP304a1, with some also detected in *S. invicta* worker antennae (Shah and Robert 2020).

### 3.4 Differential Expression Metabolome Profiles in Response to Queenright or Queenless

**FIGURE 3:**
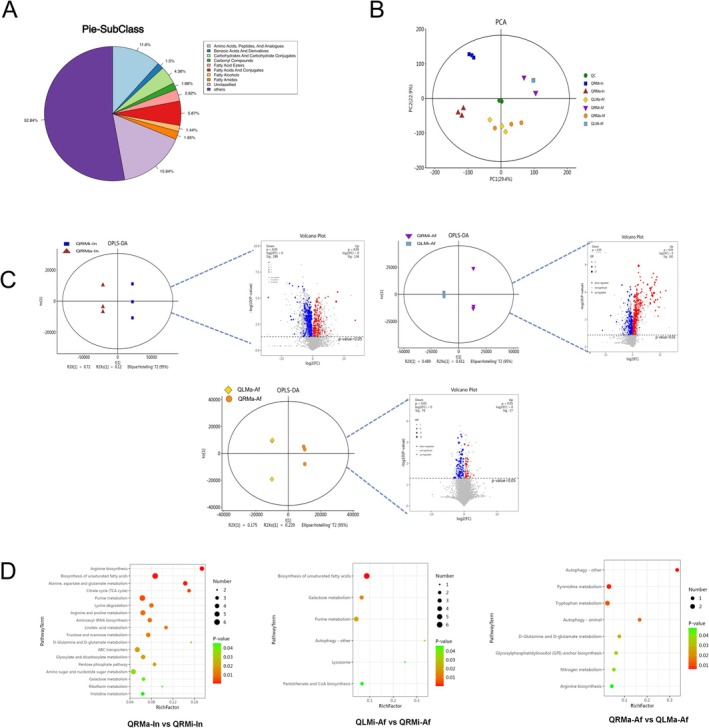
Metabolic profile changes of ant workers in queenless and queenright colonies. The figure shows the subclass distribution of worker metabolites (A) and PCA analysis for the comparisons QRMa‐In versus QRMi‐In, QLMi‐Af versus QRMi‐Af, and QRMa‐Af versus QLMa‐Af (B). OPLS‐DA and a volcano plot of differentially accumulated metabolites (DAMs) are presented (C). Significantly changed pathways based on enrichment and topology analyses are listed (D), with changes indicated at *p* < 0.05.

To investigate the physiological and metabolic effects of queen presence or absence in fire ant workers, we conducted a metabolomic analysis using LC–MS. Metabolites were categorized into nine subclasses, predominantly amino acids, peptides, analogues, and fatty acids (Figure 3A). Principal Component Analysis (PCA) and Orthogonal Partial Least Squares Discrimination Analysis (OPLS‐DA) were employed to reveal distinct metabolic patterns under queen‐right and queenless conditions. Quality control (QC) samples clustered closely, indicating the experiment's stability and repeatability (Figure 3B). A total of 981 differentially expressed metabolites were identified across the three groups, with 424 in QRMa‐In versus QRMi‐In, 454 in QLMi‐Af versus QRMi‐Af, and 103 in QRMa‐Af versus QLMa‐Af, comprising up‐regulated and down‐regulated metabolites (Figure 3C). The differentially accumulated metabolites (DAMs) were significantly up‐regulated in minor workers under queenless conditions, while both DEGs and DAMs were reduced in major workers under queen‐right conditions. KEGG database analysis revealed that DAMs were mainly enriched in pathways such as amino acid biosynthesis and metabolism, carbohydrate metabolism (including the TCA cycle), arginine biosynthesis, unsaturated fatty acid biosynthesis, and autophagy (Figure 3D). The latter two pathways showed significant enrichment under queenless conditions, aligning with transcriptomic findings and suggesting a need for further investigation into the nutritional and energy metabolic roles of fatty acids.

### 3.5 Integrated Transcriptome and Metabolome Analysis

**FIGURE 4:**
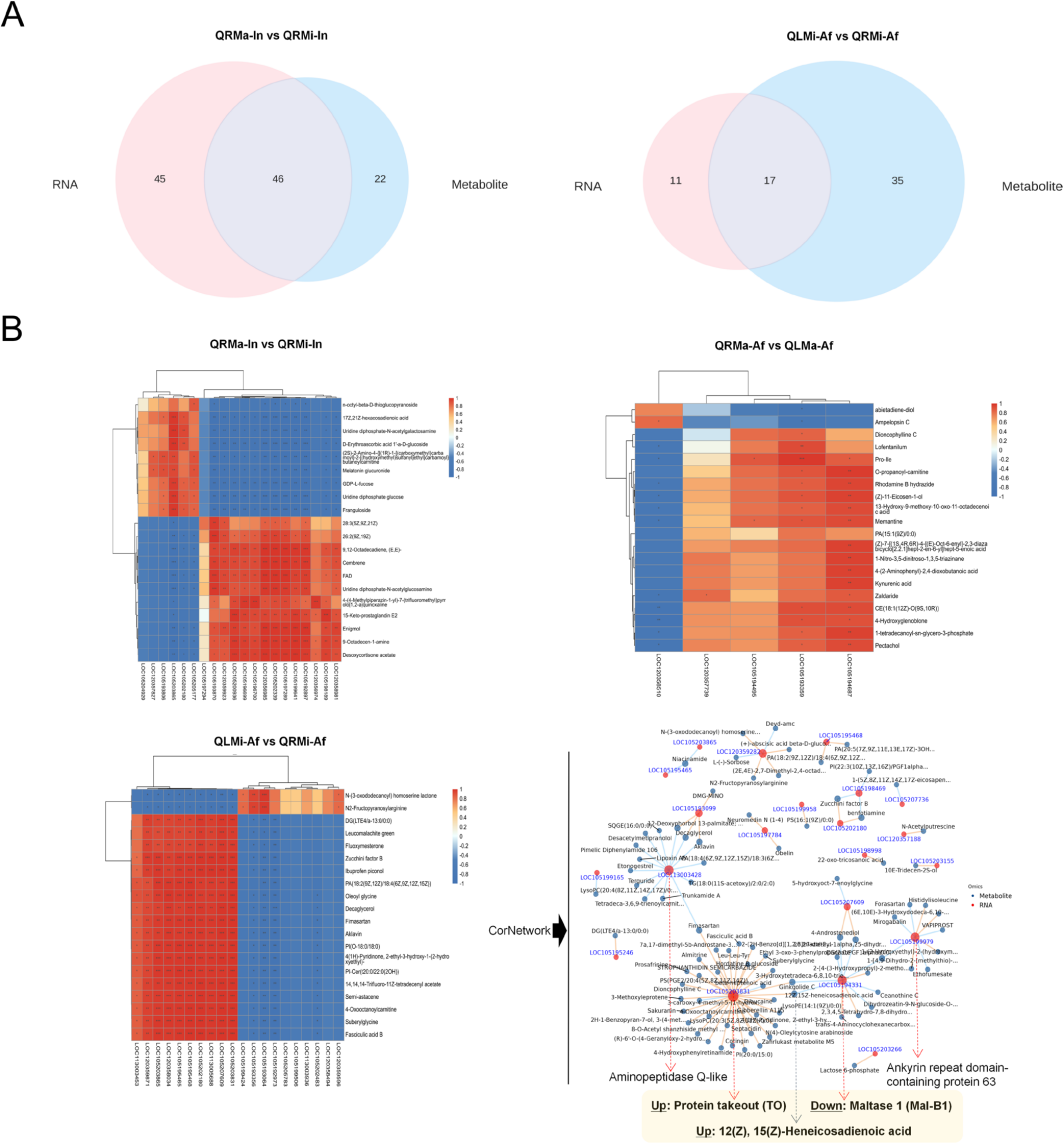
Integrative analysis of transcriptome and metabolic profiles. Overlapped pathways of differentially expressed genes (DEGs) and differentially accumulated metabolites (DAMs) are shown in a Venn diagram (A). The correlation of the top 20 DEGs and DAMs and their network in QLMi‐Af versus QRMi‐Af is depicted (B).

An integrated analysis of the transcriptome and metabolome was conducted to explore the interplay between differentially accumulated metabolites (DAMs) and differentially expressed genes (DEGs) across the study groups. Notably, 46 KEGG pathways were co‐enriched in the QRMa versus QRMi group, while 17 KEGG pathways were co‐enriched in the QLMi versus QRMi group. In contrast, the QRMa versus QLMa group exhibited minimal co‐enriched pathways (Figure 4A). The top 5 KEGG pathways identified in the QRMa versus QRMi, QLMi versus QRMi, and QRMa versus QLMa comparisons included phosphatidylinositol signaling system, glycerolipid metabolism, glycine, serine, and threonine metabolism, neuroactive ligand‐receptor interaction, and drug metabolism. The correlation analysis of the top 20 DAMs and DEGs is depicted in Figure 4B, revealing a significant association between these molecular entities. Further integration of transcriptomic and metabolomic datasets allowed us to identify co‐enriched KEGG pathways. The correlation network analysis of the QLMi‐Af versus QRMi‐Af group, focusing on the top 100 absolute values of the correlation coefficient, identified key genes with substantial influence, such as protein takeout (up‐regulated), aminopeptidase Q‐like (down‐regulated), maltase 1 (down‐regulated), and ankyrin repeat domain‐containing protein 63 (down‐regulated). Intriguingly, the metabolite 12(Z), 15(Z)‐Heneicosadienoic acid, a long‐chain polyunsaturated fatty acid, was significantly up‐regulated and correlated with the protein takeout (*TO*) and maltase 1 (*Mal‐B1*) genes (Figure 4B). The *TO* gene, which shares similarities with odorant binding proteins and juvenile hormone binding proteins, is implicated in the response to fluctuating food availability conditions (Sarov‐Blat et al. 2000).

**FIGURE 5:**
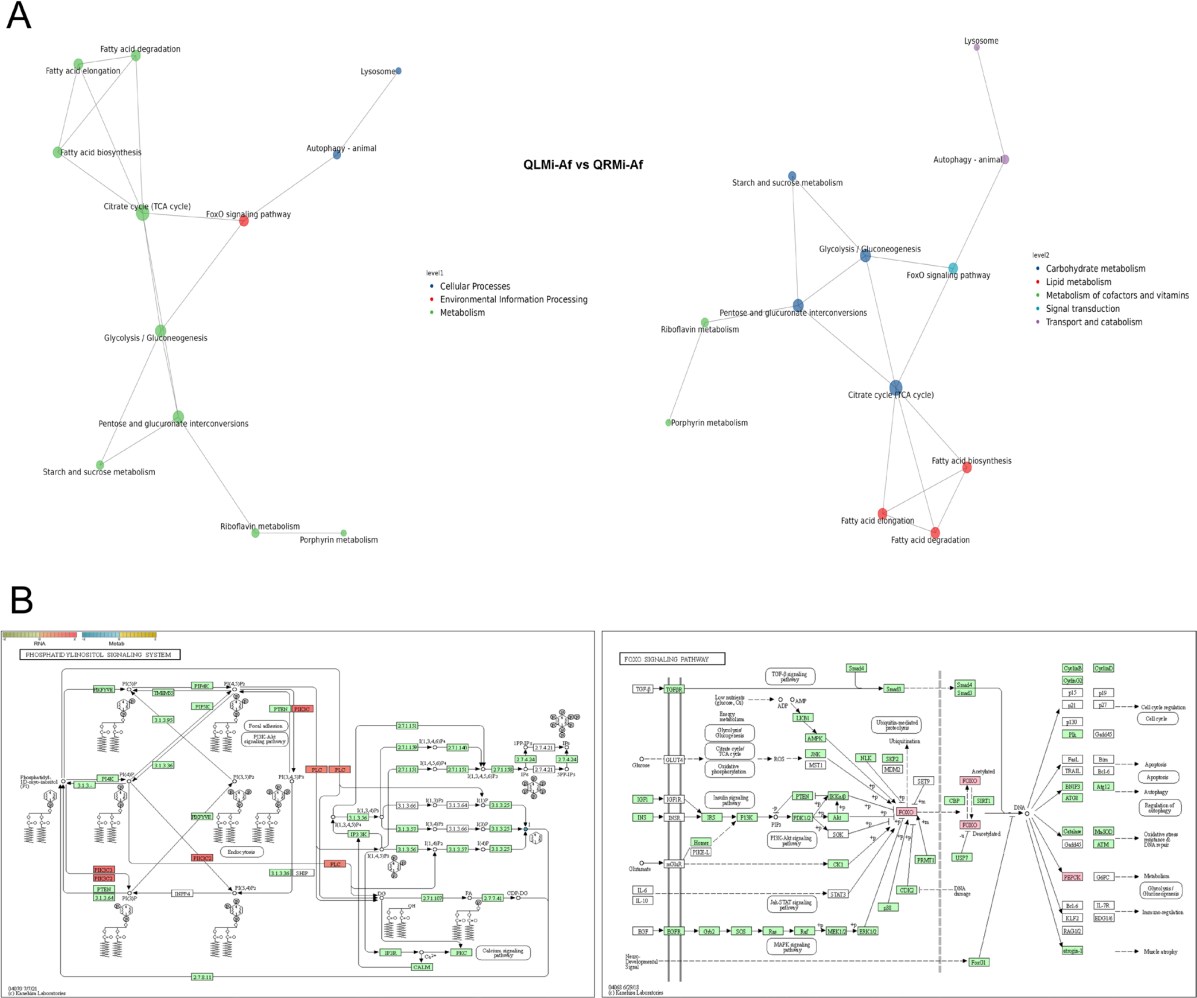
Integrative analysis of KEGG pathways. The KGML interaction network diagram of the top 30 KEGG pathways for QLMi‐Af versus QRMi‐Af is shown (A). Common enriched KEGG pathways between transcriptome and metabolic profiles are displayed, with notation for RNA (Square) and metabolism (Circle) (B).

The KGML interaction network diagram underscored the significance of the FoxO signaling pathway, the TCA cycle (protein and energy metabolism), autophagy (lysosome), and the phosphatidylinositol signaling system in the intricate interactions between genes, proteins, compounds, and pathways (Figure 5). The phosphatidylinositol and FoxO signaling pathways are pivotal for organisms to adapt to external nutritional changes, such as starvation or dietary restriction, and to regulate growth and metabolism (Tatar et al. 2014; Ebner et al. 2023). In the absence of queens, we hypothesize that worker ants initiate autophagy through the phosphatidylinositol signaling pathway in response to altered external conditions. This response involves the up‐regulation of PLC and PIK3C genes, leading to increased production of lysosomes with diverse forms and functions, such as PI(3,4,5)P. This, in turn, activates the PI3K/Akt signaling pathway, which regulates downstream transcription factors such as *FoxO* and *PEPCK* (Figure 5B).

### 3.6 Verification of Objected Genes by RT‐PCR

**FIGURE 6:**
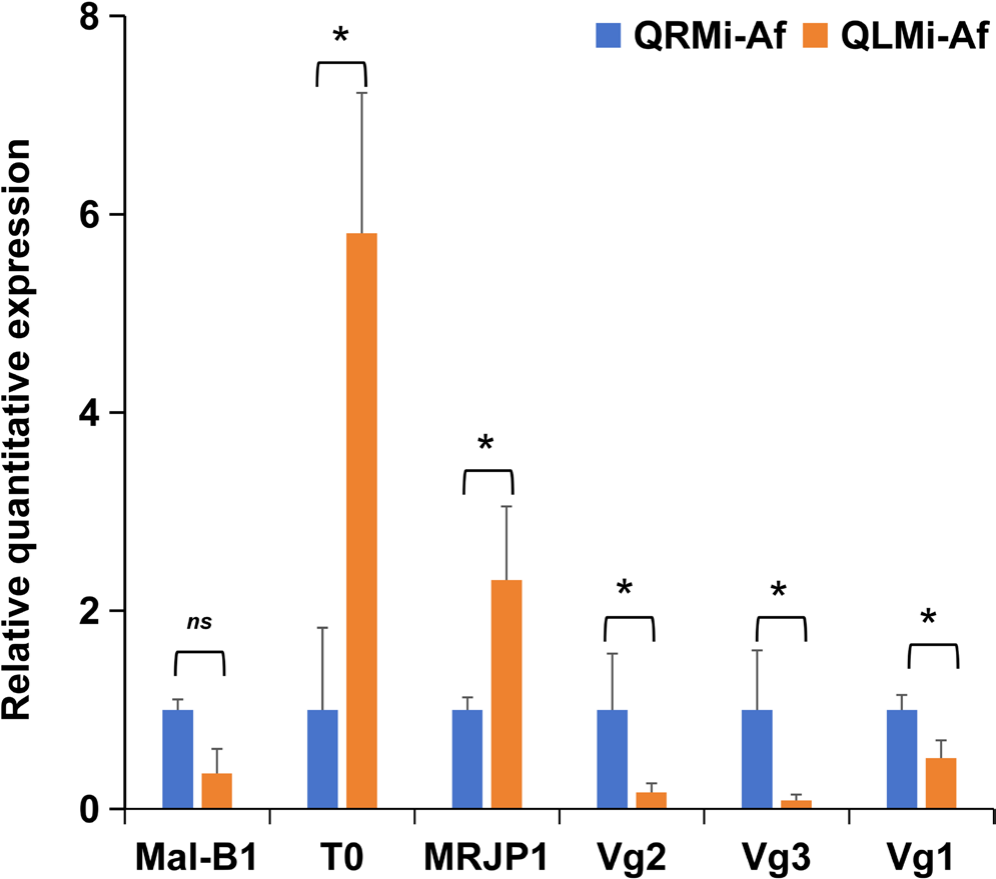
Verification of gene expression by RT‐PCR. The expression of six filtered genes (Mal‐B1, TO, MRJP1, *Vg1*, *Vg2*, and *Vg3*) in minor workers of queenless and queenright groups is verified. Bars marked with “*” indicate significant differences, while “ns” denotes no significant difference at *p* = 0.05, based on Tukey's HSD test.

We conducted RT‐PCR validation on six genes of interest to substantiate the expression patterns identified in the transcriptome analysis. These included the vitellogenin genes (*Vg1*, *Vg2*, and *Vg3*), *TO* (takeout), *Mal‐B1* (maltase 1), and MRJP1 (Major royal jelly protein 1). Our findings revealed robust expression of these genes (Figure 6). The TO protein, predominantly found in insect tissues associated with chemical sensing and nutrition, is implicated in feeding behavior, food location, and longevity (Sarov‐Blat et al. 2000). In minor workers from queenless colonies, the relative expression level of *TO* was over 5‐fold higher compared to those from queenright colonies (*p* = 0.011), suggesting significant overexpression in the absence of the queen. *MRJP1*, known for its role in ovarian development and longevity and expressed in the brain mushroom bodies of honey bees (Kamakura 2011), showed a significant increase in expression in minor workers from queenless colonies (*p* = 0.012). Additionally, in the presence of a queen, there was a substantial upregulation of *Vg1* (*p* = 0.029), *Vg2* (*p* = 0.012), and *Vg3* (*p* = 0.015) in minor workers. These vitellogenin genes are neuroendocrine targets of corazonin, which is known to regulate foraging and reproductive behaviors (Nagel et al. 2020). While the expression of *Mal‐B1* did not differ significantly between QRMi‐Af and QLMi‐Af (*p* = 0.063), there was a non‐significant trend towards decreased expression following the queen's loss (value reduced from 1 to 0.36). The downregulation of *Mal‐B1* may indicate a reduced activity in hydrolyzing maltose to glucose, potentially affecting energy metabolism in the ants.

## 4 Discussion

The phenomenon of polyethism, where all female ants in a colony share a common genome yet exhibit diverse roles, physical characteristics, behaviors, and lifespans due to environmental regulation, is well‐documented (Wilson 1987). For instance, reproductive queens can live for decades, whereas workers typically have a lifespan of only a few months. However, certain species of worker ants, such as *Harpegnathos saltator* , are capable of an extraordinary transition: upon the death or removal of the queen, they can metamorphose into reproductive pseudo‐queens with a lifespan extended up to five times (Yan et al. 2022). This study aimed to explore the behavioral, reproductive, and lifespan changes in workers of different castes following queen loss, potentially uncovering novel behavioral and physiological mechanisms.

Necrophoresis behavior, an innate nest‐cleaning activity in *S. invicta* (Howard and Tschinkel 1976), was observed to be more effective in the presence of a queen, with an increase in refuse piles by the end of the observation period. Conversely, in queenless colonies, although the number of refuse piles did not significantly change, fewer dead workers were present in the later stages of the investigation compared to queenright colonies, yet the corpses were discarded randomly. This observation suggests that queen loss may affect the worker ants' ability to recognize the corpses of their companions. Of course, this hypothesis requires further testing through assessments of the worker ants' tactile perception and recognition capabilities. Additionally, the dietary preferences of worker ants significantly altered with queen loss; the preference for white granulated sugar notably decreased by the end of the survey, while the interest in protein‐rich dried yellow mealworms remained high. Insect dietary choices, such as sugar, protein, and fat, are influenced by factors like food availability, nutrient content, and reproductive status, with precise recognition regulated by distinct taste receptor genes (Waris et al. 2024; Zhao et al. 2023). Queen loss may directly or indirectly trigger changes in taste and olfactory perception, disrupting the dietary balance. But both queenright and queenless groups decreased in the carrying rate of sugar, which may be attributed to reduced demand due to the inability to consume solid food in both cases. Then, the workers in the treatment group did not reduce the carrying of yellow mealworm carcasses containing protein and fat. This may be because the residual odor substances on the surface of yellow mealworm carcasses are also attractive to ants, or ants themselves are keen on dismembering animal carcasses. Interestingly, queen loss did not significantly affect the worker death rate compared to queenright conditions, implying an increase in worker lifespan with queen loss. Throughout the experiment, we did not observe queenless worker ants laying eggs, suggesting that the extended lifespan may not be due to reproductive regulation as previously reported (Gospocic et al. 2017; Sheng et al. 2020; Yan et al. 2022).

At the genetic level, our investigation revealed minimal transcriptional differences between queenright and queenless major workers, with only five genes differentially expressed. In contrast, minor workers exhibited more significant changes. Similar findings were reported by Manfredini et al. (2014), although the degree of differential expression between queenright and queenless minor workers was less pronounced in their study. These discrepancies may be attributed to the social form of *S. invicta* (polygyne in our case vs. monogyne) and the duration of queen loss processing (25 days in our study vs. 5 days in theirs). The authors of the previous study suggested that a 5‐day treatment might not be sufficient to observe transcriptional changes in workers (Manfredini et al. 2014). In polygyne *S. invicta* colonies, the presence of hundreds of queens may reduce the chemical communication between queens and workers (Goodisman and Ross 1997). Thus, we opted for a longer treatment duration to ensure detectable transcriptional changes. The minimal impact of queen loss on the transcriptome of major workers in our experiment could be due to their closer proximity to queens within the nest. Younger major workers, randomly paired with one of the queens in the fragmented colony, may not be as attuned to leadership changes as older foraging workers who have adapted to the colony's overall state over an extended period. However, further scientific evidence is required to validate this hypothesis.

Our study, which involved the random collection of workers from both within and outside the nest, did not distinguish between age, size, or specific division of labor. However, we identified significant gene expression differences between inside/non‐foraging major workers and outside/foraging minor workers, with 932 genes up‐regulated and 400 genes down‐regulated. Fatty acid synthesis and tissue development were found to be highly expressed in major workers, whereas hormone activity was more pronounced in minor workers. Similarly, in gamergates (formerly non‐reproductive workers), genes related to fatty acid synthesis were enriched (Yan et al. 2022). The fatty acid synthesis pathway is crucial for epidermal function and the biosynthesis of insect pheromones derived from fatty acids (Teerawanichpan et al. 2010; Tupec et al. 2017). We propose that ants active in the nest microenvironment require robust local defenses to counteract microbial infections and heightened pheromone synthesis for communication purposes. Tissue development may be linked to the size and age of workers, with the majority of nest‐based major workers anticipated to transition into foraging and defense roles, hence the up‐regulation of tissue development genes. Juvenile hormone, an endocrine hormone, is recognized for its role in regulating the division of labor in worker ants and honey bees. Amdam et al. (2006) reported higher juvenile hormone levels in foraging bees compared to nursing bees, with exogenous application accelerating the transition to foraging behavior. Additionally, octopamine, a non‐peptide neurohormone, is significant for nestmate recognition in honey bees and ants (Robinson et al. 1999; Sasaki et al. 2007). Given that foragers outside the nest are primarily responsible for nestmate recognition, we hypothesize that variations in hormone levels may correlate with differences in motility and locomotor behavior.

In our study, following a 25‐day period of queen absence, a significant down‐regulation of gene expression related to olfactory receptor activity and odorant binding was observed in minor workers. Given the high abundance of cells in workers that process olfactory information (Li et al. 2022), the queen's loss may disrupt the behavioral organization and strategy of minor workers, including their olfactory perception capabilities. The absence of the queen's primer pheromone could potentially interfere with the regulation of olfactory genes in these workers. Conversely, the gene expression of enzymes involved in fatty acid synthesis and lipid accumulation increased in minor workers post‐queen loss, possibly as a mechanism to regulate cellular balance and sustain metabolic health. The KEGG pathway analysis revealed a significant down‐regulation of cytochromes P450 in minor workers under queenless conditions. Cytochromes P450 are recognized for their role in the synthesis and degradation of endogenous compounds, such as hormones and pheromones in insects (Feyereisen 2012). Of the seven cytochrome genes identified in our study, four—CYP6k1, two variants of CYP4C1, and CYP304a1—were previously reported in the antennae of workers (Shah and Robert 2020), suggesting a role in the deactivation of odorant and pheromone molecules. CYP4g15 genes have been identified in the worker tibia (Shah and Robert 2020) and the larval gut of three moth species (Zhang et al. 2022). These genes are not only functionally conserved but are also considered ancient, originating from different bacterial species based on motif and 3D structure predictions (Zhang et al. 2022). CYP genes are believed to be insect‐specific and act as oxidative decarbonylases, playing a role in the final step of cuticular hydrocarbon (CHC) production (Balabanidou et al. 2016; Kefi et al. 2019). The up‐regulation of these genes may indirectly contribute to insecticide resistance through enhanced CHC production (Feyereisen 2020). The specific function of CYP4g15 warrants further investigation. Additionally, the CYP6a13 gene is implicated in insect resistance responses (Xu et al. 2020; Bouafoura et al. 2022), while the CYP4V2 gene, which has broad applications in human disease research (Kelly et al. 2011), is seldom reported in insects and merits future study. It has been shown that workers exhibit the highest levels of CYPABs mRNA expression across different developmental stages and castes of *S. invicta* (Liu and Zhang 2004). In our study, the down‐regulation of cytochrome P450‐related genes in minor workers post‐queen loss primarily impacts the deactivation of odorant and pheromone molecules. We propose that queen loss directly impacts the olfactory behavior of minor workers, which could subsequently affect their learning and cognitive abilities and may even indirectly influence their dietary choices.

**FIGURE 7:**
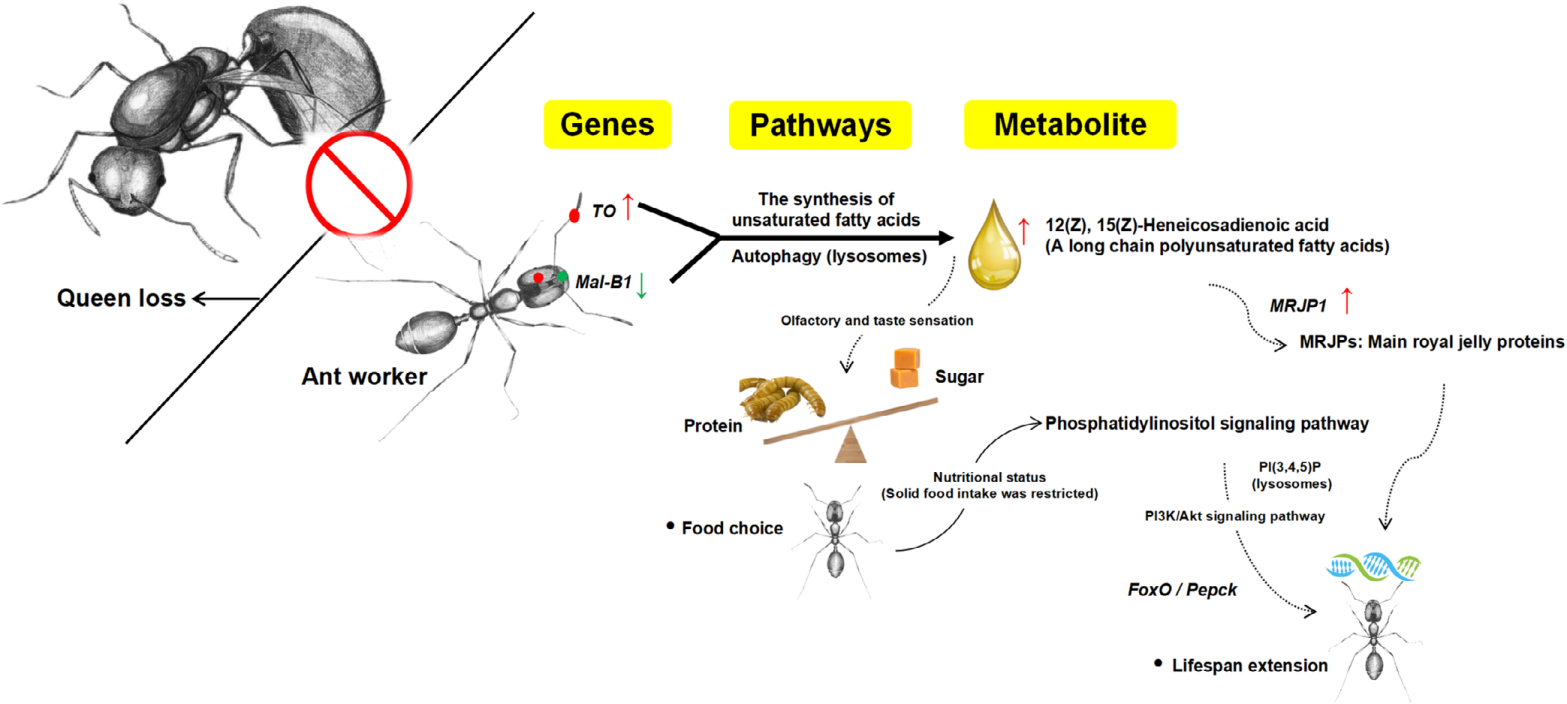
Molecular regulatory network of minor‐worker ants dominated by *TO* gene in the absence of queen loss.

Our integrated analysis of the transcriptome and metabolome revealed significant roles for two influential genes, *TO* and *Mal‐B1*, and their associated metabolite, (12(Z), 15(Z)‐heneicosadienoic acid). The *TO* gene is integral to the chemosensory systems of insects, involved in taste and olfaction (Sarov‐Blat et al. 2000; Bohbot and Vogt 2005), with its expression linked to food intake and affecting various digestive systems and antennal structures. Notably, this gene's expression is heightened by starvation, enhancing the sensitivity of taste neurons to sugars in *Drosophila* and thus promoting food consumption (Meunier et al. 2007). The results indicate a marked increase in *TO* gene expression within the QLMi‐Af group. However, we observed a significant reduction in sugar transport during different nutritional status, suggesting a potential regulatory role of the *Mal‐B1* gene in sugar intake, possibly due to its down‐regulation. Given that minor workers experienced dietary restriction in the absence of a queen, we hypothesize that the *TO* gene may be up‐regulated in the ants' brains and antennae. The *Vg* gene is a pivotal regulator of reproductive development in ants; its expression in the fat body of *Harpegnathos* workers surges as they transition into queens (Yan et al. 2022). Conversely, we noted a significant decrease in *Vg* gene expression in minor workers post‐queen loss, implying no reproductive activity in this scenario or a shift towards foraging behaviors (Nagel et al. 2020). Although no reproductive activity was detected in minor workers without a queen, we did observe an extension of their lifespan. We attribute this to the significant up‐regulation of the *MRJP1* gene in minor workers, which is responsible for the production of major royal jelly proteins (MRJPs). These proteins, known for their role in ovarian development, hormonal balance, and longevity in honey bees (Kucharski et al. 1998; Drapeau et al. 2006; Buttstedt et al. 2014), are thought to be key nutrients that determine the development of queen honeybees (Kamakura 2011). While their role in ants is less documented, we propose that MRJPs may positively influence longevity in queenless conditions. Given that MRJPs are lipid‐rich and primarily composed of fatty acids, the significantly up‐regulated (12(Z), 15(Z)‐heneicosadienoic acid) may be implicated in MRJP synthesis, presenting an avenue for further research. Isolated workers, free from queen pheromones, may have their longevity influenced by the colony environment, particularly in response to pathogens and dietary constraints (Ye et al. 2019). We propose that the longevity extension in minor workers without a queen could be distinct from previously reported mechanisms linking reproduction and longevity, such as those involving insulin (Yan et al. 2022), catalase (Orr and Sohal 1994; Ye et al. 2019), or parasite mediation (Hartke et al. 2023). Instead, the extension may be attributed to the combined effects of queen pheromone absence and nutritional status, mediated by the *TO* and *MRJP1* genes (Figure 7).

In our study, the KGML interaction network diagram illuminated significant enrichment of pathways in the biosynthesis of unsaturated fatty acids and autophagy, particularly in queenless conditions. Additionally, the FoxO and phosphatidylinositol signaling pathways were pivotal in mediating interactions among genes, proteins, compounds, and pathways. Prior research indicates that the activation of autophagy and FoxO signaling pathways can facilitate the clearance of cellular damage, provide energy, and extend lifespan under conditions of starvation or dietary restriction (Tatar et al. 2014; Ebner et al. 2023). Autophagy is triggered when cellular nutrients or energy are scarce, leading to the degradation of minor proteins and redundant organelles by lysosomes to supply necessary materials and energy (Luzio et al. 2007; Ohsumi 2014). Lysosomes, as key organelles in autophagy, enable organisms to sense and rapidly respond to fluctuations in external nutritional status through the autophagy/lysosome pathway (Ebner et al. 2023). For instance, during starvation, the lysosomal localization of the synthetase PI4K2A increases, catalyzing the production of more lysosomal PI(4)P, which replaces the activation of *mTOR* and ultimately promotes the catabolism of lipids and proteins (Ebner et al. 2023). Phosphatidylinositol signaling on lysosomes is crucial for regulating lysosomal morphology and function (Balla 2013; Tan and Finkel 2022). We hypothesize similar metabolic and regulatory networks in our study's context (Figure 7). Minor workers without a queen initiated autophagy through the phosphatidylinositol signaling pathway, up‐regulating *PLC* and *PIK3C* genes, and producing a variety of lysosomes with different functions, such as PI(3,4,5)P. This action stimulates the PI3K/Akt signaling pathway. Activation of the downstream transcription factor *FoxO* within this pathway likely induced a cascade of lipid formation and decomposition processes in the fat body (Hossain et al. 2013; Li et al. 2019), potentially extending lifespan by regulating nutrition. Hormones in the brain are implicated in worker ants' foraging behavior, development, and reproductive regulation. The *TO* gene, as previously highlighted, may trigger this series of expressions and the production of key metabolites (Figure 7). Our study not only demonstrates that *S. invicta* workers lack the potential to transform into queens but also offers a novel model for uncovering the molecular mechanisms behind changes in worker longevity without accompanying shifts in reproductive capacity. In the future, further in‐depth research can be conducted on the roles of epigenetic mechanisms such as DNA methylation and histone modification in the regulation of the lifespan and reproductive capacity of worker ants. For example, epigenetic profiles of worker ants in different lifespan and reproductive states could be analyzed to identify differentially modified sites and clarify how these modifications influence gene expression and cellular functions. Additionally, comparative analyses between *S. invicta* and other ant species could be performed to uncover evolutionary principles among ant species by examining similarities and differences in their epigenetic mechanisms.

**Table:** 

		5 days		25 days	
		QR	QL	QR	QL
Refuse pile	1	4	3	6	3
	2	3	2	5	3
	3	3	4	3	4
	4	4	2	5	2
	5	2	2	3	3
	6	3	4	4	4
	7	3	3	5	4
	8	3	3	4	3
	9	4	5	5	5
	10	3	2	5	3
	11	4	3	4	3
	12	3	3	5	4
Food transportation: dried yellow mealworm	1	3	3	4	7
	2	3	3	4	6
	3	2	2	5	5
	4	3	4	6	5
	5	3	3	7	5
	6	4	3	10	6
	7	5	2	7	8
	8	4	1	5	4
	9	3	2	6	7
	10	4	3	7	7
	11	5	1	6	5
	12	5	3	5	5
Food transportation: white granulated sugar	1	20	12	33	15
	2	15	17	31	10
	3	17	14	27	17
	4	18	19	36	23
	5	20	17	22	10
	6	20	18	21	9
	7	18	18	17	6
	8	17	15	32	11
	9	16	17	36	17
	10	19	19	33	10
	11	20	15	30	9
	12	15	11	25	9

		25 days		45 days	
		QR	QL	QR	QL
Number of corpses	1	19	14	72	34
	2	38	21	93	49
	3	14	17	57	37
	4	10	10	65	28
	5	31	28	88	46
The initial number of ants	1	304	310	304	310
	2	324	308	324	308
	3	318	312	318	312
	4	308	312	308	312
	5	322	302	322	302

## Author Contributions

**Jun Huang:** conceptualization (equal), data curation (equal), investigation (equal), methodology (equal), writing – original draft (equal). **Shizhen Wang:** data curation (equal), formal analysis (supporting), investigation (supporting), methodology (equal), writing – original draft (supporting). **Chendi Yu:** data curation (supporting), formal analysis (supporting), methodology (equal), writing – original draft (equal). **Hongli Su:** formal analysis (supporting), investigation (equal), methodology (supporting), resources (supporting). **Zhitao Jiang:** data curation (supporting), methodology (supporting), software (equal), visualization (equal). **Xiaowei Li:** formal analysis (supporting), writing – original draft (supporting). **Yaobin Lu:** conceptualization (supporting), funding acquisition (equal), project administration (equal), resources (supporting), supervision (supporting), writing – review and editing (equal). **Juan Zhang:** conceptualization (supporting), funding acquisition (equal), project administration (equal), validation (supporting), writing – original draft (equal), writing – review and editing (equal).

## Conflicts of Interest

The authors declare no conflicts of interest.

## Supporting information


Data S1.

Data S2.

## Data Availability

Raw data generated in this study were submitted to the NCBI Sequence Read Archive database and MetaBoLight database, under the accession number: PRJNA1112102 and unique identifier: MTBLS11541, respectively. Data will be made available on request.
