# Supplementary material for: Multi‐Omics Analysis Reveals TO Gene's Association With Food Selection and Lifespan in Minor‐Worker Ants Post‐Queen Loss
Source: Ecol Evol. 2025 Jun 7;15(6):e71508. doi: 10.1002/ece3.71508 (PMC12144595; doi:10.1002/ece3.71508)

**Supplementary Material**

**Supplementary Table 1** The primers used in during RT-PCR

| Gene NAME | Forward primer(5->3) | Reverse primer(5->3) |
| --- | --- | --- |
| EF1-beta | TGAAGACCGATAAGGGCATC | TTCCCCAGTGCCTCAAATAC |
| Mal-B1 | TGTATCCCGTGATCCTGAAC | GCCAGATTTAACGTCTTGTAG |
| T0 | GCAACTGGGTTATCCAGAAG | TGAGACCAGTTATTCGCAGG |
| MRJP1 | TCGATTGCGTTAGAAGGTCC | AACGTTCGCACACATAATAG |
| Vg2 | GCTATCTTAGCTGAAGATGCG | TCGACATTCTGTCCGTTTACT |
| Vg3 | TGCAACATATCGTAATGCCA | GGTTTCGTCAAGAGACAGTTC |
| Vg1 | GGTACTAAGAATATGGCGACT | GTTCCTTTGTATGCACTATACG |

**Supplementary Figure S1** Ant behavior testing and observation

**
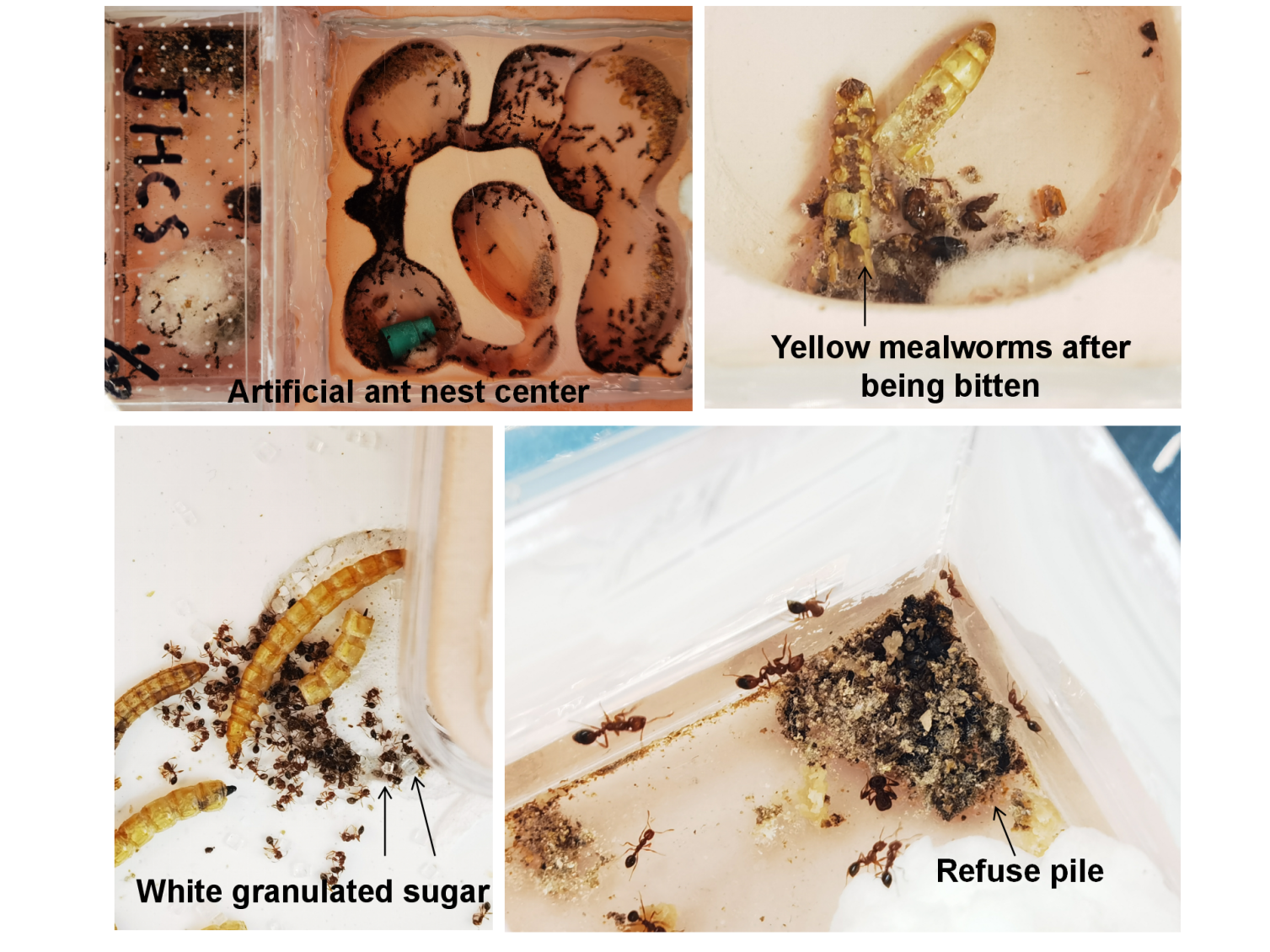
**

**Supplementary Figure S2** Ant collection and rearing condition


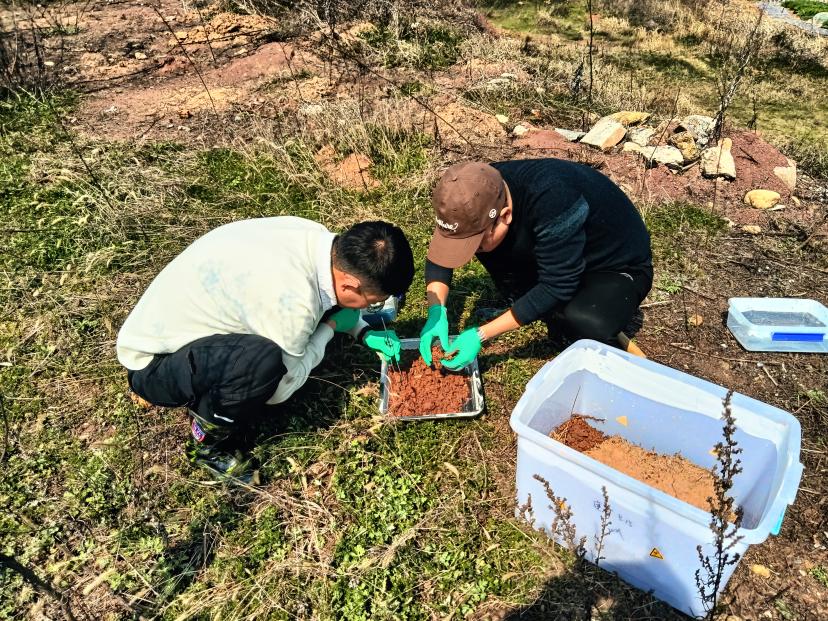

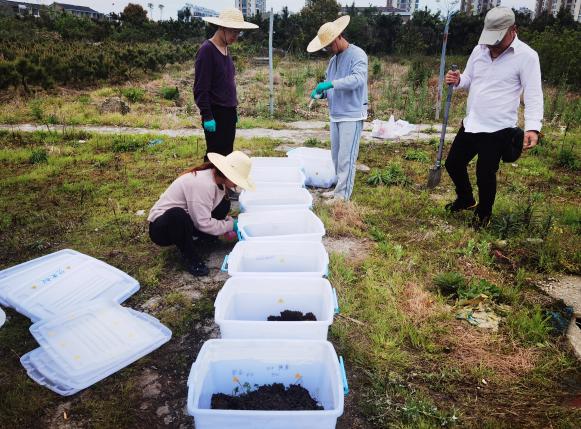


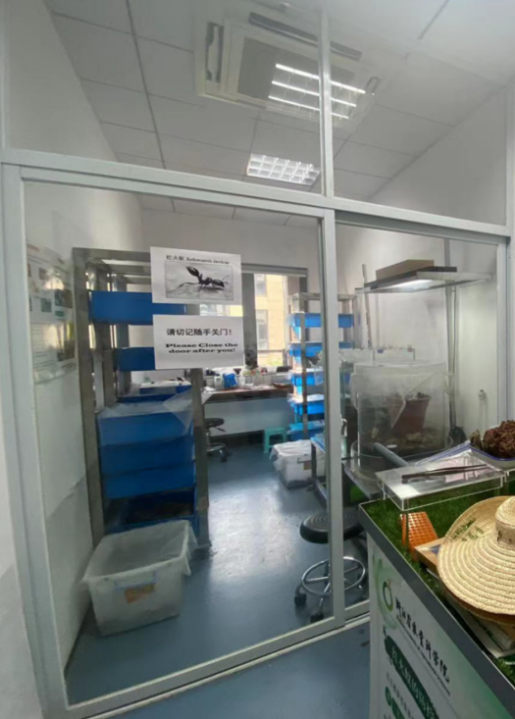

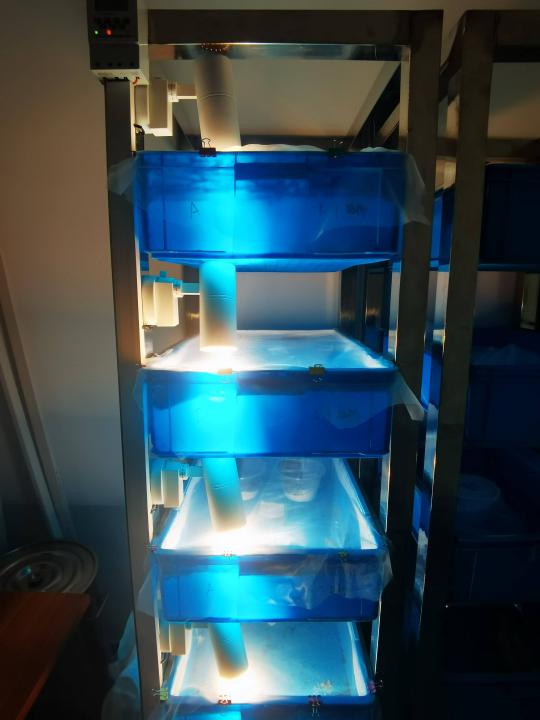

Supplement: Supplementary file 2 — Data S2. [file ECE3-15-e71508-s002.docx]
